# Use of the rSpaA415 antigen indicates low rates of *Erysipelothrix rhusiopathiae* infection in farmed cattle from the United States of America and Great Britain

**DOI:** 10.1186/s12917-019-2147-7

**Published:** 2019-11-01

**Authors:** Ana I. Cubas Atienzar, Priscilla F. Gerber, Tanja Opriessnig

**Affiliations:** 1 The Roslin Institute and The Royal (Dick) School of Veterinary Studies, University of Edinburgh, Easter Bush, Midlothian, UK; 2 Animal Science, School of Environmental and Rural Science, University of New England, Armidale, Australia; 3 Department of Veterinary Diagnostic and Production Animal Medicine, College of Veterinary Medicine, Iowa State University, Ames, Iowa, USA

**Keywords:** *Erysipelothrix rhusiopathiae*, Cattle, Prevalence, US, Great Britain, Growth agglutination test, Fluorescent microbead-based immunoassay, Enzyme-linked immunosorbent assay

## Abstract

**Background:**

Clinical cases of *Erysipelothrix rhusiopathiae*, a zoonotic gram-positive bacterium, have been reported in many ruminant species, including in cattle, deer, moose and muskoxen. Fatal cases have been repeatedly reported in cattle over the years but to date there is only one Japanese study investigating the seroprevalence of this bacterium in cattle using the growth agglutination test (GAT). This technique is subjective, time-consuming, expensive and hazardous compared to modern serological tests such as enzyme-linked immunosorbent assays (ELISA) or the newly developed fluorescent microbead-based immunoassays (FMIA).

**Results:**

The FMIA based on the surface protein SpaA (rSpaA415) antigen of *E. rhusiopathiae* developed in this study had an almost perfect agreement with the GAT (k = 0.83) and showed a sensitivity of 89.7% and a specificity of 92.9% when compared to the GAT. Overall, detection rates of *E. rhusiopathiae* antibody positive samples were 13.8% (51/370) in British herds and 6% (12/200) in US herds. Positive cattle were present in 34.3% (24/70) of the investigated British farms and in 34.7% (8/23) of the US farms with an on-farm prevalence of 7.1 to 100% for the British farms and 8.3–30% for the US farms.

**Conclusions:**

FMIA is a fast, safe and economic alternative to the GAT for the diagnosis of *E. rhusiopathiae* in cattle. This work is the first seroprevalence study of *E. rhusiopathiae* in healthy farmed cattle in Great Britain and the US and revealed that infection occurs at a low level. Further investigations to evaluate risks of zoonotic transmission when handling cattle are needed.

## Background

*Erysipelothrix* is a gram-positive bacterium. The genus *Erysipelothrix* contains four relevant species associated with 28 different serotypes; *Erysipelothrix rhusiopathiae* (serotypes 1a, 1b, 2, 4, 5, 6, 8, 9, 11, 12, 15, 16, 17, 19, 21, 23 and N), *Erysipelothrix tonsillarum* (serotypes 3, 7, 10, 14, 20, 22, 24, 25 and 26), *Erysipelothrix sp.* strain 1 (serotype 13) and *Erysipelothrix sp.* strain 2 (serotype 18) [1]. *Erysipelothrix rhusiopathiae* is considered to contain pathogenic isolates known as the etiologic agent of swine erysipelas associated with sporadic cases or larger outbreaks of major economic importance [2]. Besides pigs, *E. rhusiopathiae* can cause a wide range of diseases in other species such as sheep, fish, poultry, cattle and humans [3–6]. Infections in humans are primarily a result of contact with infected animals and are presented either as a localized cutaneous lesion called erysipeloid, as a generalised cutaneous lesion, or as a septicaemic form which is often associated with endocarditis [7].

Recently, *E. rhusiopathiae* has been isolated in increasing frequency from ruminants, especially from farmed cattle (*Bos taurus*) [4, 5]. Most of the clinical cases in cattle are observed in young calves presenting septicaemia [8], with abscesses in the liver and lungs [9], encephalomeningitis [10], or polyserositis and arthritis [11].

*Erysipelothrix rhusiopathiae* has been associated with unusual mortality events in muskoxen (*Ovibos moschatus wardi*) in the Canadian Arctic Archipelago [12]. During the years 2009–2011, a total of 22 muskoxen were found dead during expeditions in the Canadian Arctic Archipelago and in 2012 approximately 150 muskoxen were found dead; *E. rhusiopathiae* serotype 5 was confirmed by serotyping isolates from tissues of these animals [12]. Interestingly, *E. rhusiopathiae* serotype 5 was also isolated from a fatal case of metritis in a Norwegian heifer [13] and from a fatal case of acute multifocal necrotic hepatitis in a white tailored reindeer in Iowa, USA [14]. In Canada, the death of three elks (*Alces alces*) was linked to a septicaemia caused by *Erysipelothrix* of serotype 17 [15].

During studies in Japanese abattoirs, *Erysipelothrix* was isolated from 6.4% of 1236 healthy, slaughtered cattle [16] which demonstrates that cattle may be subclinically infected with the bacterium. A follow-up epidemiological study using the growth agglutination test (GAT) to detect anti-*Erysipelothrix* antibodies in Japanese cattle found that 76% of 854 healthy cattle had detectable antibodies [3]. The same study also found a higher rate of seropositive cattle in areas also having swine industry [3]. This data could indicate that *Erysipelothrix* is mainly transmitted by pigs although cattle may also act as a vehicle for its distribution [5, 16]. In support of this, *Erysipelothrix* was isolated from cattle slurry [3] which could enhance the bacterium’s ability to spread as *Erysipelothrix* can survive in soil contaminated with faecal material [4].

Previously studies investigating antibodies in cattle have been carried out using solely GAT. GAT has been extensively used in pigs and chickens and it has shown a good correlation between the antibody titres and immune status in vaccinated pigs [17] and challenged chickens [18] but this correlation has not yet been investigated in cattle. The use of GAT in pigs and chickens was replaced by developed enzyme-linked immunosorbent assays (ELISAs) and fluorescent microbead-based immunoassays (FMIAs) [6, 19–22] due to their ability to permit the testing of large numbers of samples in a short time, while giving objective results. FMIAs are based on a liquid suspension array designed for multiplex testing. This technology utilizes magnetic microspheres filled with a distinct red and infrared fluorescent dyes, resulting in up to 100 sets of different microspheres each of which with its own unique spectral address allowing heavy multiplexing in one reaction well.

Although *Erysipelothrix* and antibodies against it have been detected in healthy cattle in Japan [3–5], data is lacking for the distribution of *Erysipelothrix* in cattle across Europe and North America where its epidemiological importance is not known.

A highly sensitive (96.5%) and specific (100%) ELISA was recently developed for the detection of *Erysipelothrix* in swine using a recombinant SpaA (rSpaA415) [6]. This assay was then further improved by adapting it into an FMIA [21]. Compared to the ELISA, the FMIA is more sensitive and its format requires less serum, less antigen and allows multiplexing thereby further reducing cost.

This study aimed to investigate the antibody distribution against *Erysipelothrix* in cattle in Great Britain and the US to increase the knowledge of its epidemiological significance and to develop an ELISA and FMIA test using rSpaA415 antigen for the detection of antibodies against *Erysipelothrix* in cattle to overcome the disadvantages of GAT.

## Results

### Development and optimisation of rSpaA415 FMIA and ELISA and cut-off evaluation using the GAT as the reference assay

A first subset of 300 samples were tested with the ELISA and FMIA to evaluate the performance of both tests. The FMIA had superior performance compared to the ELISA. Specifically, the FMIA had an area under the curve (AUC) of 94.8% (95% CI, 91–98%) indicating a high accuracy of the test. The optimal cut-off was determined to be 1073.5 MFI giving a diagnostic sensitivity and specificity of 89.7 and 92.9% respectively. The ELISA had an AUC of 64.7% (95% CI, 55–74%) and a sensitivity and specificity of 61 and 53% for the optimal cut-off which had an S/*P* value of 22%.

ELISA and FMIA had an agreement of 79% and a kappa value of 0.31 showing a fair agreement. FMIA was preferred because it was more accurate, sensitive, specific, and easy to operate than the ELISA and was selected to test the remaining field samples. The percentage of agreement of the FMIA and GAT was 93% (95% CI, 88–96%) which was almost perfect (κ = 0.83). McNemar’s test showed that the paired discordances occurred randomly and therefore, both tests were comparable (McNemar’s *p* value = 1). The CVs obtained for the FMIA test for the positive (13.1%) and negative (14%) reference sera were considered good suggesting a high level of assay reproducibility.

### Antibody titres on field and experimental samples

**Fig. 1:**
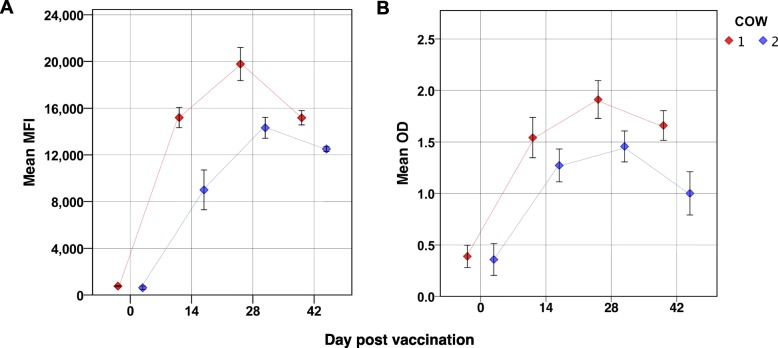
Erysipelothrix rhusiopathiae antibody levels in vaccinated cows. Median fluorescent intensity (MFI) (**a**) and ELISA optical density (OD) (**b**) values before vaccination (day 0) or at day post vaccination (dpv) 14, 28 and 42 days. Vaccination was done using an *Erysipelothrix rhusiopathiae* vaccine on dpv 0 and 14. Values represent the average of three repeats (+/− 2 SE)

Experimentally vaccinated cattle developed antibodies against *E. rhusiopathiae* as demonstrated by the FMIA, ELISA and GAT (Fig. 1, Table 1). Both cows had detectable antibodies on 14 dpv and antibody levels remained detectable until the 42 dpv. MFI and GAT titres were higher in experimentally vaccinated cattle compared to non-vaccinated field cattle with natural exposure. The distribution of GAT titres against *E. rhusiopathiae* for the studied bovine samples is summarized in Table 1. The majority of positive field samples had GAT titres of 32 (22%, 36/162), followed by 64 (13%, 21/162) and 128 (13%, 5/16). The majority of the samples tested showed agglutination below the cut-off (50%, 81/162). High antibody titres were detected more frequently in British cattle compared to US cattle (Table 1).

**Table 1:** Distribution of growth agglutination test (GAT) *Erysipelothrix rhusiopathiae* antibody titres (cut-off 1:32) in cattle of unknown *Erysipelothrix* spp. status (British and US herds), cows experimentally vaccinated (VAC), or in gnotobiotic calves

GAT titre	0	1:4	1:8	1:16	1:32	1:64	1:128	1:256	1:512	1:1024	Total
British herds	8	12	12	34	30	17	5				112
US herds	12	14	3	6	6	4					50
VAC cow 1			dpv 0						dpv 14	dpv 42, 28	4
VAC cow 2		dpv 0						dpv 14	dpv 28, 42		4
Gnotiobiotc	10										10

*Dpv* day post vaccination with an *Erysipelothrix rhusiopathiae* vaccine

**Fig. 2:**
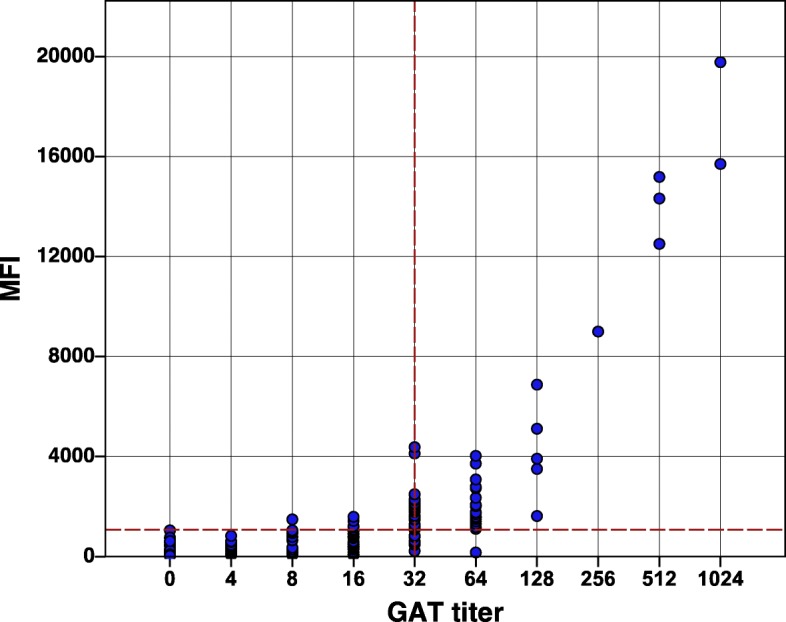
Distribution of Erysipelothrix rhusiopathiae titers in field and experimental samples (*n* = 181). Vertical dashed line represents the growth agglutination test (GAT) cut-off (1:32) and the horizontal line the fluorescent microbead-based immunoassay (FMIA) cut-off (1073.5)

MFI and GAT titres were plotted and showed a good correlation (Fig. 2) which was supported by Pearson coefficient (*r* = 0.93).

### *Erysipelothrix* antibody rates in US and British cattle herds by FMIA

Antibodies against *E. rhusiopathiae* were present in both populations of farmed cattle at a low level. Overall, the prevalence in the studied British cattle population was 13.8% (51/370). Positive cattle were identified in 34.3% (24/70) of the farms tested with an on-farm prevalence of 7.1 to 100%. The number of seropositive cows among the sampled US population was 6% (12/200), positive cattle were identified in 34.7% (8/23) of the investigated farms, and the on-farm prevalence ranged from 8.3–30%. The difference in the prevalence between the US and Great Britain was statistically significant by Chi-square test (*p* = 0.0047, χ^2^ = 7.99) and the difference in prevalence between farms was statistically significant for British farms (*p* = 0.002, Fisher value = 86.5) but not for US farms (*p* = 0.23, Fisher value = 18.9).

## Discussion

The GAT was used as the reference test as it is the only methodology used so far to evaluate the *Erysipelothix* antibody responses in cattle. The FMIA developed in this study is an improved tool for the detection of *Erysipelothix* antibodies in cattle and had almost perfect agreement with the GAT, with a high sensitivity and high specificity. There are three main disadvantages of the GAT. First, the assay is time consuming as only 8 samples can be included in a plate with two overnight incubations, one to prepare the inoculum and a second one to read the results. Second, the GAT is hazardous since live pathogenic bacteria have to be used. Finally, and third, the results are read by naked eye which may be subjective and variable between operators. In contrast, the FMIA permits the testing of large numbers of samples in less than 4 h and gives precise and objective results. For these reasons, we propose in here the use of FMIA as an alternative to GAT.

The specificity of the GAT in cross-reaction with antibodies against organisms other than *E. rhusiopathiae* has not been studied in detail. The cross-reactivity of rSpaA415 has been validated with pig pathogens but it remains unknown for pathogens infecting cattle. Higher specificity is predicted by using FMIA than GAT as the GAT involves an extent of non-specific agglutination since a titre of less of 32 indicates a negative reaction.

Differences in sensitivity and specificity between GAT and FMIA could be due to the *Erysipelothrix* serotypes infecting cattle. The serotype used in the GAT is serotype 1a but it is uncertain to what extent the GAT agglutinates across different *E. rhusiopathiae* strains or other *Erysipelothrix* species. Antibody responses against rSpaA415 were detected in sera from rabbits inoculated with *E. rhusiopathiae* serotypes containing Spa A (1a, 1b, 2, 5, 9, 12, 15, 16, 17 and 23), Spa B1 (4, 6, 8, 19 and 21), Spa B2 (11) and *E. sp.* strain 2 containing Spa C (serotype 18). In contrast, low antibody responses were observed in sera from rabbits inoculated with *E. tonsillarum* serotype 20 (no Spa type) and no antibody response was observed in sera from rabbits inoculated with the remaining serotypes without Spa type (*E. tonsillarum* and *E. sp.* strain 1) [6]. The knowledge of the *Erysipelothrix* serotypes infecting cattle is limited; one study in Japan recovered 79 *Erysipelothrix* isolates from the tonsils of healthy slaughtered cattle of which only 43 out of these isolates were typeable and were classified into the serotypes 1b, 2, 3, 5, 9, 12, 13, 19 and 21. Responses against rSpaA415 were detected in rabbits infected with serotypes 1b, 2, 5, 9, 12, 19 and 21 but not with serotypes 3 and 13 [6]. There is no data of the serotypes of the *Erysipelothix* serotypes circulating among the US and British herds but in Norway, a serotype 5 was isolated from an heifer with fatal metritis [13].

This study showed that FMIA was more sensitive and specific than the ELISA using the same antigen. Higher sensitivity of FMIA assays than ELISA have been reported in other studies [21, 23, 24]. Higher sensitivity of the FMIA in comparison to the ELISA using the rSpaA415 protein was observed in experimentally infected pigs, where the FMIA could detect more pigs with an *Erysipelothrix* IgG antibody response than the ELISA at day 7 post challenge. The overall sensitivity in experimentally infected pigs was 94.4% for the FMIA and 73.6% for the ELISA [21].

The higher specificity of FMIA assays has been suggested due to proteins covalently coupled to microspheres, and antigen purity eliminates nonspecific reactions that often cause high background problems in ELISA [25]. The lower background may increase the detection limit on samples with a low antibody concentration [21].

Because there are no studies outside Japan that have investigated the seroprevalence of *E. rhusiopathiae* in bovine samples, we considered this knowledge gap important. Although the number of samples is limited to extrapolate the prevalence obtained in this study with the general prevalence in UK and US herds, this work represents the first study investigating *E. rhusiopathiae* in British and US cattle and the results suggest that *E. rhusiopathiae* infections in cattle occurs at low likely subclinical levels. All the animals sampled in this study were healthy and had no clinical signs, suggesting that as reported before [3, 16], asymptomatic cattle could be carrying the bacterium.

Although the percentage of farms with seropositive cows was nearly the same in both countries; the overall prevalence in the sampled British herds (13.8%) was significantly higher than in the US (6%) and positive British cattle had higher antibody titres when compared to the studied US herd. The reason for this is not clear; in Japan, higher cattle antibody rates were obtained where there were also areas of swine industry [3]. This association could not be studied in the present work, since the information about the location and biosecurity measures of the sampled farms was not provided. It is worthy to mention however, that in the most common type of farming in the UK cows are kept on pasture during the grass growing seasons while this may not be the case in the US. The outdoor access and the biosecurity measures intrinsically associated with this type of farming could be an explanation for the higher prevalence in the British cattle as animals would have more chance to get in contact with the bacterium than US cattle. Nevertheless, this conjecture needs further investigation as there are not studies that compare the prevalence on different farming systems and also, we do not know the location of the farms sampled in the study and thus we have no manner to support this hypothesis.

Challenge studies in pigs have shown that a titre above 32 in the GAT indicates protective immunity against infection but it is unknown whether this applies also to cattle. The cattle vaccinated with the pig vaccine showed much higher antibody titres (GAT 256–1024, MFI 10,000-19,000) than the *E. rhusiopathiae* positive unvaccinated field cows (GAT 32–128, MFI 1074–6800).

Seroprevalence studies in other ruminant species besides cattle are virtually absent; only one Japanese study found a seroprevalence of *Erysipelothix* of 13.5% among 52 wild deer (*Cervus nippon yesoensis* and *Cervus nippon centralis*), also using the GAT [26].

The obtained results suggest that 13.8 and 6% of the farmed British and US cattle have had exposure to *Erysipelothrix* spp. and could be carriers of the bacteria and be a potential source of infection for other animals and humans. Furthermore, 34.4 and 34.7% of the sampled farms in Great Britain and the US had at least one seropositive animal. This data is of epidemiological importance as the bacterium has been isolated from cow slurry [3] and it is very resistant to environmental challenges [4], being able to survive in contaminated soil for several months [8]. Preventive measures should be implemented to avoid the transmission of the pathogen, especially among, abattoir workers, butchers, farmers and veterinarians that are at higher risk of exposure [27].

## Conclusions

This work is the first one investigating the seroprevalence of *E. rhusiopathiae* among North American and British cattle and results showed that, although at low frequency, cattle are exposed to the bacterium and therefore could act as a reservoir of transmission of the disease. The newly developed FMIA test represents an improvement on the serodiagnosis of this bacterium in cattle as it is fast, proving objective results, sensitive (89.7%), specific (92.9%) and has a good agreement with the reference assay GAT.

## Methods

### Serum samples

#### Cattle with known *Erysipelothrix* exposure

Two adult Angus feedlot cattle, part of the Iowa State University teaching herd and located in Ames, IA, USA were vaccinated with a commercial vaccine licensed for pigs (MaGESTic® 7 with SPUR®; Intervet Inc., DE, USA, serial number: 0784A009A) twice with 14 days interval by injecting 2 ml of the vaccine intramuscular into the neck area. Blood samples were collected before vaccination (day 0) and at days 14, 28 and 42 post vaccination (dpv), centrifuged, and the serum was collected and used for further analysis. The two cattle remained in the teaching herd after the experiment ended. In addition, 10 archived serum samples from gnotobiotic calves from an unrelated study (kindly provided by Dr. Geraldine Taylor, Pirbright Institute, Surrey, UK) were used as controls.

#### Field samples

A total of 200 US feedlot cattle serum samples submitted for routine diagnostics were obtained from the Veterinary Diagnostic Laboratory at Iowa State University, Ames, IA, USA (kindly provided by Dr. David Baum) and 370 serum samples were obtained from Scottish dairy cattle through the Scottish Agricultural College (SAC), Midlothian, UK (kindly provided by Dr. Jill Thomson). All animals were at least 1 year old (adults), healthy and had no clinical signs. The serum samples corresponded to 23 US farms with an average of 8.6 animals per farm (min = 1, max = 15, median = 10) and 70 Scottish farms with an average of 5.3 animals per farm (min = 1, max = 15, median = 3).

### Serological assays

#### ELISA and FMIA development

The recombinant protein rSpaA415, based on the major surface protective antigen A (SpaA) [6, 21] was used as antigen in both ELISA and FMIA. The optimal dilution of the serum sample and regents was determined by a checkerboard titration in both assays.

For the FMIA, 18 μg of the polypeptide rSpaA415 was coupled to 5 million of microbeads (bead region 45, Luminex Corp., TX, USA) using a 2 step carbodiimide reaction [21]. All the incubations were carried out for 30 min at room temperature, in the dark and under continuous shaking. Washing steps were performed using a magnetic separator and 200 μl of washing buffer composed of PBS with 0.05% of Tween 20, pH 7.4 (PBST).

Coupled beads were diluted in blocking buffer (StabilGuard; Surmodics, MN, USA) to a final concentration of 2500 beads per well and 50 μl of this solution were incubated with 50 μl of each serum sample diluted 1:800 in assay buffer (PBST containing 10% goat serum). The plate then was incubated and washed three times with washing buffer. Following this, 50 μl of a 20,000 dilution of a biotin conjugated goat anti-bovine IgG (Jackson Immuno Research, Cambridge, UK) was added to the wells and incubated for another 30 min. After three washings, 50 μl of streptavidin R-phycoerythrin conjugate (SAPE; MOSS, MD, USA) at 1:100 dilution in assay buffer were added to the wells. Finally, after the last incubation and washing steps, beads were re-suspended in 100 μl of assay buffer and analysed using a MAGPIX® reader system (Luminex corp., TX, USA).

The in-house ELISA was performed in 96 well plates (MaxiSorp™; Nunc, NY, USA) coated with 0.3 μg/ml of rSpaA415 polypeptide and blocked using PBS with 10% chicken serum (Biowest, MO, USA) for 2 h at room temperature. The samples were diluted at 1:800 with PBS containing 10% rabbit serum (Biowest, MO, USA) and 100 μl was added to each well of the plate and left to incubate for 30 min at 37 °C. After washing the plates, 100 μl of a 1:30,000 dilution of peroxidase-conjugated rabbit anti-bovine (Jackson Immuno Research, Cambridge, UK) was added to each well and left to incubate for 30 min at 37 °C. After the final washing, 100 μl of tetramethylbenzidine-hydrogen peroxide (TMB) was added to each well as a substrate (KPL, MD, USA) and left for 15 min for colour to develop. The peroxidase reaction was stopped by adding 50 μl of 1% HCl solution (KPL, MD, USA) into each well. The optical density was then read at 450 nm using a spectrophotometer.

#### Growth agglutination test

The growth agglutination test was conducted to determine the agglutinating antibody titres of the sera as described elsewhere [3, 17, 18]. Briefly, 50 μl of each serum sample was incubated for 1 h at 37 °C with 50 μl of 0.2 M 2-Mercaptoethanol (Sigma Aldrich, MO, USA). Two-fold dilutions (1:2 to 1: 2048) of the serum were made in brain heart infusion broth supplemented with 0.1% Tween 80 (BHI-T80), 0.3% Tris-HCl (pH 8.0), kanamycin (100 μg/ml) and gentamicin (50 μg/ml) in a 96 well plate. Following this, 50 μl of the *E. rhusiopathiae* strain E1-6P (serotype 1a), diluted 1:10 after an 18 h incubation in BHI-T80, was added to each of the wells. Agglutination reactions were read after incubation at 37 °C for 18 h. The titres were expressed as the reciprocal of the number of the highest dilution of serum that showed agglutination.

### Data analysis

Statistical evaluations were performed with the package SPSS (v.19). Statistical significance was set at a *p* value of < 0.05. The level of agreement between tests was determined using Cohen’s Kappa and the percentage of agreement. Pearson coefficient was used to measure the level of correlation between tests. Kappa coefficients (k) with values between 0 and 0.01, 0.02–0.2, 0.21–0.4, 0.41–0.6, 0.61–0.8 and 0.81–1 were interpreted as no agreement, slight, fair, moderate, substantial and almost perfect agreement respectively [28]. Marginal homogeneity of paired data was tested by McNemar’s Chi-square test [29]. Assay results were expressed as Median Fluorescent Intensity (MFI) for FMIA and Optical Density (OD) and S/*P* value calculated as the (average OD sample/OD positive reference control) × 100 for the ELISA. The inter-assay variance was calculated using the coefficient of variance (CV) for a positive and a negative. CV with values < 10, 10–15%, 15–20%, > 20% were considered excellent, good, acceptable and excessive. A set of field samples (*n* = 163) were chosen based on MFI and OD values and all available controls (*n* = 18) and GAT was used as reference test to classify samples as positive or negative and MFI and S/*P* value were analysed in a ROC curve to generate an optimised cut-off [30].

## Data Availability

All data generated during this study is presented in an analysed format is this manuscript. Raw datasets generated during the current study are available from the first author (ana.cubasatienzar@lstmed.ac.uk) on reasonable request.

## Abbreviations

BHI: Brain heart infusion broth
CI: Coefficient interval
CV: Coefficient of variance
ELISA: Enzyme-linked immunosorbent assay
FMIA: Fluorescent microbead immunoassay
GAT: Growth agglutination test
HCl: Hydrochloric acid
IgG: Immunoglobulin G
MFI: Medium fluoresce intensity
OD: Optical density
PBS: Phosphate-buffered saline
PBST: PBS with 0.05% of Tween 20
PCR: Polymerase chain reaction
ROC: Receiver operating characteristic
SE: Sensitivity
SP: Specificity
T80: Tween 80
TMB: Tetramethylbenzidine-hydrogen peroxide
κ: kappa coefficient
